# Speciation and Thermodynamic Study of Arsenic(III)–Pharmaceutical Complexes in Aqueous Solutions

**DOI:** 10.1021/acsenvironau.5c00024

**Published:** 2025-05-12

**Authors:** Federica Carnamucio, Claudia Foti, Franz Saija, Giuseppe Cassone, Ottavia Giuffrè

**Affiliations:** † Department of Pharmaceutics and Center for Pharmaceutical Engineering and Sciences—School of Pharmacy, Virginia Commonwealth University, 410 N 12th Street, Richmond, Virginia 23284, United States; ‡ Dipartimento di Scienze Chimiche, Biologiche, Farmaceutiche ed Ambientali, 18980 Università di Messina, Viale F. Stagno d’Alcontres 31, Messina 98166, Italy; § Institute for Chemical-Physical Processes, 9327 National Research Council of Italy (CNR-IPCF), Viale Ferdinando Stagno d’Alcontres 37, Messina 98158, Italy

**Keywords:** metronidazole, nalidixic acid, As(III), speciation study, density functional theory

## Abstract

Background: Natural water sources are increasingly contaminated with a wide range of pollutants including heavy metals and pharmaceuticals. Arsenic, particularly in its more toxic trivalent form, i.e. As­(III), remains a significant environmental and public health concern due to its widespread presence and carcinogenic effects. In addition to that, pharmaceutical products like metronidazole (MNZ) and nalidixic acid (NAL), persistent in the environment due to their limited biodegradability, also pose significant threats to both ecosystems and human health. Recent research has highlighted the formation of antibiotic-metal complexes (AMCs) where antibiotics interact with heavy metals in aquatic environments, leading to altered physicochemical properties and increased toxicity. Aim: The main objective of the present work is a speciation study on As­(III)–antibiotic complexes and particularly interaction between As­(III) and MNZ or NAL in aqueous solution. Methods: Several temperatures and ionic strengths were probed by potentiometry to determine the formation constants and other thermodynamic parameters of As­(III)–MNZ and As­(III)–NAL complexes. UV spectrophotometric titrations were also employed to confirm formation constants of both systems. An estimation of the sequestering ability of both ligands toward As­(III) under relevant natural water conditions has also been performed. Further, density functional theory calculations have been executed with the purpose of investigating the molecular structure of these complexes and their relative stability. Results: It turns out that MNZ binds to As­(III) in either a neutral (AsMNZ) or protonated (As­(MNZ)­H) form via As–N and As–O interactions, with the hydroxyl oxygen being the preferred binding site in AsMNZ and both the nitro and hydroxyl groups being equally effective in As­(MNZ)­H, while NAL forms a stable chelated complex through bidentate coordination. Conclusion: Findings reported in this study contribute to a deeper understanding of the complexes formed by As­(III) with pharmaceuticals and pave the way toward the development of improved technologies for the water treatment and remediation of AMCs.

## Graphical abstract

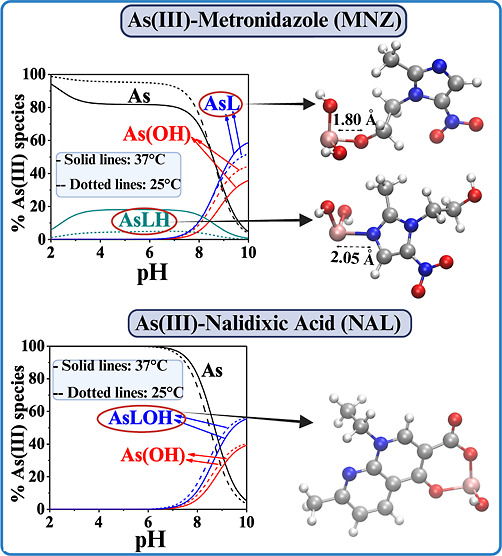

## 1 Introduction

Natural water sources have a wide range of pollutants, including heavy metals, chemicals, pharmaceuticals such as antibiotics, pathogens, pesticides, fertilizers, microplastics, and personal care products.^1^ Among these contaminants, heavy metals, especially arsenic, pose significant environmental and health risks.^2^ Arsenic, a toxic and ubiquitous element, is commonly found in water in two valence states: arsenite (As­(III)) and arsenate (As­(V)).^3^ Of these, As­(III) is considered the most hazardous one due to its extreme toxicity and carcinogenic properties.^4^ Chronic exposure to As­(III)-contaminated water has been linked to liver damage, as well as an increased risk of bladder and lung cancer, cardiovascular ailments, cancer, and skin blemishes.^4^ Furthermore, studies have demonstrated that arsenic can disrupt antioxidant systems in human red blood cells, amplifying its cytotoxic effects.^5,6^ The global prevalence of arsenic contamination in drinking water is alarming, with groundwater samples from over 70 countries reporting arsenic concentrations far exceeding the World Health Organization’s recommended limit of 10 μg L^–1^.^3,6–9^ Reports have shown arsenic concentrations in groundwater ranging from 0.5 to 5000 μg L^–1^, raising significant public health concerns, particularly in developing countries where water treatment infrastructure may be inadequate.^10,11^ Even low-level exposure to arsenic has been linked to chronic diseases, including cancer, cardiovascular problems, and neurological disorders.^12–14^

Beyond arsenic and heavy metals, pharmaceuticals such as metronidazole (MNZ) and nalidixic acid (NAL) (Figure 1) pose significant environmental and human health risks due to their persistence in the environment and their toxicity.^15–18^ These pharmaceuticals, which are widely used to treat bacterial infections in both humans and animals, often end up in aquatic environments due to improper disposal, agricultural runoff, or inefficient removal during wastewater treatment.^19^ The environmental persistence of MNZ and NAL, coupled with their high solubility in water and low biodegradability, makes them particularly concerning contaminants in natural water sources.^19^ Studies have reported the detection of both antibiotics in rivers, lakes, groundwater, and even drinking water, indicating their widespread occurrence in aquatic ecosystems.^19^ Their presence can have harmful effects on aquatic organisms and may contribute to the growing problem of antimicrobial resistance, which poses a significant global health threat.^9^ All these aspects have led to their classification as contaminants of emerging concern (CEC).^9^

**Figure 1:**
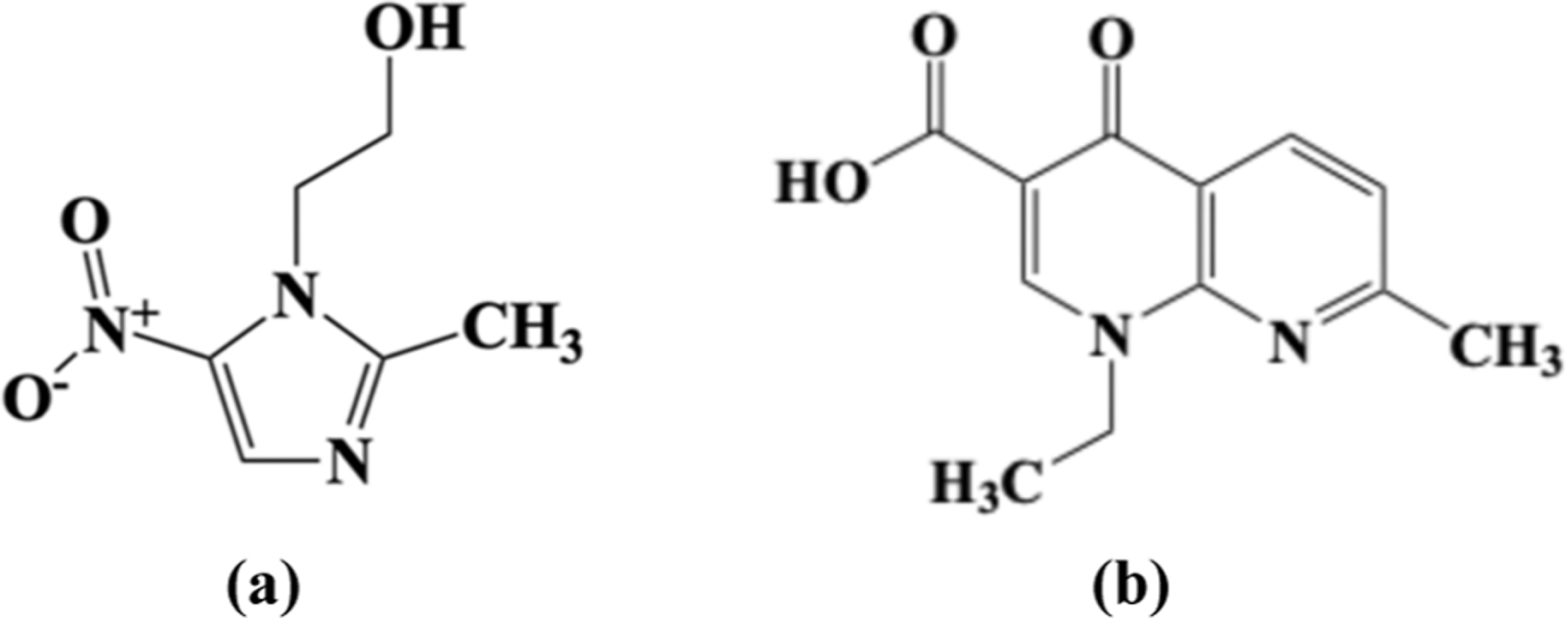
(a) Metronidazole (2-methyl-5-nitroimidazole-1-ethanol, MNZ) and (b) nalidixic acid (1-ethyl-7-methyl-4-oxo-[1,8]­naphthyridine-3-carboxylic acid, NAL).

A particularly concerning class of pollutants is the combination of antibiotics with metal ions, leading to the formation of antibiotic-metal complexes (AMCs).^20,21^ In natural waters, where both heavy metals and antibiotics coexist, significant metal–antibiotic complexation can occur.^22^ The physico-chemical properties and biological activities of these complexes differ markedly from those of the individual antibiotics.^23,24^ AMCs tend to exhibit higher toxicity, greater persistence, and altered reactivity, making their environmental behavior more complicated.^25^ Factors such as temperature, pH, ionic strength, and the presence of natural organic matter influence the formation and stability of these complexes.^26^ AMCs also disrupt microbial ecosystems, inhibit, or encourage the growth of specific algae, fungi and bacteria, and promote the spread of antibiotic resistance genes.^21^ The complexation process also affects the bioavailability, absorption, solubility, and photolysis of the antibiotics, further complicating their environmental impact.^27,28^ These various forms may have different reactivity, toxicity, mobility, persistence, bioavailability, and potential ecological impact in aquatic ecosystems affecting their behavior in the environment and the effectiveness of removal methods.^2,9,29–36^ Given that both MNZ and NAL contain functional groups that can interact with metals, such as As­(III), they can form AMCs. Therefore, studying the speciation of these AMCs is essential for understanding their behavior, potential ecological risks, and long-term environmental impacts. Despite the increasing recognition of the significance of AMCs, the interaction between As­(III) and pharmaceuticals such as MNZ and NAL remains poorly understood, particularly under natural water conditions.

The most common remediation methods for AMCs include adsorption, ion exchange, precipitation, and oxidation–reduction reactions, with adsorption using materials like activated carbon and metal–organic frameworks (MOFs).^11^ However, these methods are costly and often generate toxic waste.^11^ Decomplexation, an emerging method, involves using advanced oxidation processes (AOPs) to break the bonds between heavy metals and ligands by generating reactive oxygen species, which decompose organic pollutants and release metals for further removal through adsorption or ion-exchange.^10^ Despite its potential, decomplexation has limitations due to the poor understanding of AMC speciation under varying environmental conditions, such as pH and temperature, which complicates the decomplexation process and limits the optimization of treatment methods.^37^ Further research into AMC speciation is needed to improve the effectiveness and sustainability of these approaches for environmental remediation. To address this knowledge gap, the current study investigates the formation constants and thermodynamic parameters of As­(III)–MNZ and As­(III)–NAL complexes,^38–41^ using potentiometry, UV spectroscopy, and density functional theory (DFT) calculations. More in detail, the speciation models and stability constants were obtained for both systems by UV spectrophotometric and potentiometric titrations at *t* = 25°C and *I* = 0.15 mol L^–1^ on solutions containing different As­(III)–MNZ and As­(III)–NAL concentrations and ratios. Moreover, potentiometric titrations were also performed at other temperature values (*t* = 15, 25, 37°C) and ionic strength (*I* = 0.15, 0.5, 1 mol L^–1^) in NaCl aqueous solutions to study the dependence of formation constants of these species on temperature and ionic strength. The molecular structures of the metal complexes and their relative stability will also be discussed based on DFT calculations.

In summary, this study is designed to provide a deeper understanding of the interactions between As­(III) and emerging pharmaceutical pollutants like MNZ and NAL. By investigating the speciation and thermodynamic properties of these complexes, this research aims to enhance our ability to develop more effective methods for water treatment and environmental remediation, addressing the pressing issue of contaminated water sources worldwide.

## 2 Materials and Methods

### 2.1 Materials

Solutions of As­(III) were obtained by dissolving (meta)­arsenite salt (Sigma-Aldrich, ≥90%) and standardized by titration with EDTA solution (ethylenediamine tetraacetic acid disodium salt, Sigma-Aldrich, purity ≥99%). Ligand solutions were prepared by weighing and dissolving metronidazole salt (Alfa-Aesar/Thermo Fisher, purity ≥97%) and nalidixic acid sodium salt (Sigma-Aldrich, purity ≥99%). Solutions of hydrochloric acid and sodium hydroxide were obtained by dilution of Fluka ampules, subsequently standardized with dried sodium carbonate (Sigma-Aldrich, purity ≥99.5%) and potassium biphthalate (Sigma-Aldrich, purity ≥99.5%), respectively. Solutions of sodium chloride (supporting electrolyte) were obtained by weighing the corresponding salt (Sigma-Aldrich, puriss.), previously dried in an oven at 110 °C. Distilled water (conductivity < 0.1 μS cm^–1^) and grade A glassware were employed to prepare all solutions.

### 2.2 Potentiometric Apparatus and Procedure

Two distinct systems were employed for the potentiometric titrations, consisting of an identical configuration of an automatic dispenser Metrohm Dosino 800, a Metrohm model 809 Titrando potentiometer, and a Metrohm LL-Unitrode WOC combined glass electrode. Each potentiometric system was connected to a PC and the experimental titration data were acquired by Metrohm TIAMO 2.2 software. Estimated accuracy of both systems is ±0.15 mV for emf and ±0.002 mL for the titrant volume. Each titration involved additions of volumes of NaOH standard to 25 mL of As­(III), ligand (MNZ or NAL), HCl, and NaCl under stirring to homogenize the solution. Experimental details on potentiometric titrations are reported in Table 1. For all measurements, glass-jacketed thermostated cells were used to perform titration under different temperature conditions (15 ≤ *t*/°C ≤ 37). In addition, pure N_2_ was bubbled through the solutions to remove CO_2_ and O_2_ inside the solutions. For each measurement, an independent titration of HCl with standard NaOH in a very wide pH range was performed to calibrate the electrode, thus enabling the calculation of the standard electrode potential *E* ^0^ and the p*K*_w_ value under consistent experimental ionic strength and temperature conditions.

**Table 1:** Experimental Conditions for Potentiometric and Spectrophotometric Titrations

technique	ligand	*t*/°C	*I*/mol L^–1^	*C*_As_/mmol L^–1^	*C*_L_/mmol L^–1^	M/L ratio	pH range
potentiometry	MNZ	15	0.16	1–2	1–4	0.33–1	2–10
		25	0.16	1–2	1–4	0.33–1	2–10
		25	0.50	1–2	1–4	0.33–1	2–10
		25	0.97	1–2	1–4	0.33–1	2–10
		37	0.16	1–2	1–4	0.33–1	2–10
	NAL	15	0.15	0.5–1	0.5–1	0.75–1.5	3–10
		25	0.16	0.5–1	0.5–1	0.75–1.5	3–10
		25	0.50	0.5–1	0.5–1	0.75–1.5	3–10
		25	0.98	0.5–1	0.5–1	0.75–1.5	3–10
		37	0.15	0.5–1	0.5–1	0.75–1.5	3–10
spectrophotometry	MNZ	25	0.15	0.04–0.1	0.05–0.08	0.33–1	2–10
	NAL	25	0.15	0.02–0.03	0.03–0.05	0.33–1	3–10

### 2.3 UV–Vis Apparatus and Procedure

The spectrophotometric titrations were carried out by a Varian Cary 50 UV–Vis spectrophotometer equipped with an optical fiber with a fixed 1 cm path length, interfaced to a PC with the Varian Cary WinUV software. For each titration point, the couple data of absorbance (Abs) and pH vs volume of titrant (mL) were recorded simultaneously by using a Methrom glass electrode and a Metrohm-Titrando 809 potentiometer. All titrations were performed using a thermostated cell to keep the desired temperature under N_2_ to remove CO_2_ and O_2_ from the solutions. The experimental conditions of the titrations are reported in Table 1. For each system, at least four measurements were performed scanning the spectral range of 225 ≤ λ/nm ≤ 400 in different conditions of the As­(III)/ligand ratio. Before each measurement, a baseline containing only HCl, NaCl, and H_2_O was recorded to subtract the matrix contribution.

### 2.4 Postprocessing Calculations

The most reliable speciation model for each metal–complex system under study, the formation constant values of the species, and the parameters of each titration (standard potential *E* ^0^, analytical concentration of the reagents, junction potential) were determined by processing potentiometric data by using the BSTAC4 and STACO4 programs. The parameters for the dependence of complex formation constants on temperature were obtained by LIANA program. More details on software employed in the refinement of the experimental data are reported in a study by Gianguzza et al.^42^ HypSpec program^43^ was used for UV data, enabling to refine protonation and formation constants as well as the molar absorption coefficient values (ε) of all species. HySS program was used to obtain the speciation diagrams and the formation percentages of the complex species.^44^ The sequestering ability of both ligands toward As­(III) was evaluated by using the program ES4SEQ2.^45^

### 2.5 Quantum-Mechanical Calculations

Quantum-mechanical calculations were performed by means of the Gaussian 09 software.^46^ This latter, exploiting DFT, allowed identification of the ground-state molecular structures of several complexes formed by both MNZ and deprotonated NAL with [As­(OH)_*x*_]^*y*^ (*x* = 1,2; *y* = 2+,1+) complexes and evaluation of the chelation sites and the respective binding energies of closely related relevant structures. As it is clear, different charge states were sampled. In this work, all calculations were performed using the B3LYP^47–49^ hybrid exchange and correlation functional, with 100% of exact exchange. Geometry optimization of the molecular structures was performed by employing the 6–311++G­(d,p) atomic basis sets for all atoms by employing tight convergence criteria for electron density. As far as the simulation of the solvent is concerned, the conductive polarizable continuum model (CPCM)^50^ was employed by setting parameters mimicking the water electrostatics (i.e., dielectric constant). After structural relaxation to the ground state, vibrational calculations were performed not only to establish the correctness of the previous calculations (i.e., absence of imaginary frequencies for stationary points associated with potential energy minima) but also to obtain the zero-point energy associated with each optimized molecular structure. Nuclear quantum effects have to be taken into account carefully in proton transfer phenomena because of their relevance in water also under ambient conditions.^51,52^

## 3 Results and Discussion

### 3.1 As­(III)–MNZ and As­(III)–NAL Complexes

Potentiometric titrations were conducted to investigate the interaction between As­(III) and the ligands MNZ and NAL in aqueous solutions by varying As­(III) and ligand (L) concentrations, As­(III)/L ratios, temperature, and ionic strength conditions. Previously determined protonation constants of both ligands^53,54^ were used in this study (Table S1 of Supporting Information). As is well-known, in aqueous solution, As­(III) is found in the form As­(OH)_3_ but, for the sake of simplicity, it will be denoted as As throughout the manuscript. The hydrolytic constants of As­(III) species are detailed in Supporting Information (Table S2).

The most reliable speciation models were obtained by running a series of calculations based on several criteria, such as simplicity, species formation percentages, statistical parameters (variance, variance ratio, and mean deviation), literature data, and similarity of the model referring to similar systems. For example, for As­(III)–MNZ system, Table 2 reports some trials for the selection of the speciation model from the analysis of experimental potentiometric data together with the variance ratio. The best speciation models were found with two species, namely, MLH and ML in the As­(III)–MNZ­(L) system, and AsLOH in the As­(III)–NAL­(L) system, with respective overall formation constant values listed in Table 3. To better evaluate the stability of the species, results are also reported as stepwise formation constants, i.e., log*K* (As­(OH)_3_ + H_*i*_L).

**Table 2:** Some Trials for the Selection of the Speciation Model from the Analysis of Experimental Potentiometric Data

L	species	*t*/°C	*I*/mol L^–1^	logβ^a^	logβ^a^	*t*/°C	*I*/mol L^–1^	logβ^a^	logβ^a^	logβ^a^
MNZ	AsLH	15	0.16	14.61(3)^b^		25	0.16	13.76(10)^b^		
	AsL			6.06(3)	5.67(4)^b^			5.63(2)	5.40(2)^b^	5.40(3)^b^
	AsL_2_									–6.68(12)
				σ^2^/σ_0_^2^ = 1^3^	σ^2^/σ_0_^2^ = 1.3^c^			σ^2^/σ_0_^2^ = 1^c^	σ^2^/σ_0_^2^ = 1.2^c^	σ^2^/σ_0_^2^ = 1.3^c^

a β refers to the reaction: As + L + *i*H = AsLH_*i*_ (charges omitted for simplicity).

b ≥95% of confidence interval.

c Variance ratio σ^2^/σ_0_^2^ (σ^2^ = variance of the fit, σ_0_^2^ = variance for the best fit).

**Table 3:** Experimental Overall Formation Constants of As­(III)–MNZ­(L) and As­(III)–NAL­(L) Species Obtained by Potentiometry

L	species	*t*/°C	*I*/mol L^–1^	logβ^a^	log*K* ^b^
MNZ	AsLH	15	0.16	14.61(3)^c^	2.61(3)^*c*^
		25	0.16	13.76(10)	2.09(10)
		25	0.50	14.11(6)	1.60(6)
		25	0.97	14.42(7)	2.20(7)
		37	0.16	13.20(10)	1.32(10)
	AsL	15	0.16	6.06(3)	6.06(3)
		25	0.16	5.63(2)	5.63(2)
		25	0.50	5.95(3)	5.95(3)
		25	0.97	5.89(7)	5.89(7)
		37	0.16	5.15(5)	6.06(5)
NAL	AsLOH	15	0.15	–5.82(5)	3.44(5)
		25	0.16	–6.13(5)	2.97(5)
		25	0.50	–5.98(9)	3.05(9)
		25	0.98	–5.83(6)	3.20(6)
		37	0.15	–5.93(10)	2.99(10)

a β refers to the reaction: As + L + *i*H = AsLH_*i*_ (charges omitted for simplicity).

b *K* refers to the reaction: As + LH_*i*_ = AsLH_*i*_ (charges omitted for simplicity).

c ≥95% of confidence interval.

The distribution diagram in Figure 2a shows that at *t* = 25°C, the AsL species forms within the pH range of 7.5–10, reaching a maximum of 60% at pH 10. In contrast, the AsLH species is present across the entire pH range of 2–10, with its highest formation percentage (20%) occurring between pH 3.5 and 8. When the temperature is increased to *t* = 37°C, the formation percentages of both species decrease to approximately 30% for AsL and 5% for AsLH. Figure 2b illustrates that at *t* = 25°C, the AsLOH species forms within the pH range of 7.5–10, reaching a maximum of 30% at pH 10. When the temperature is raised to *t* = 37°C**,** the formation of this species increases to 40% at the same pH.

**Figure 2:**
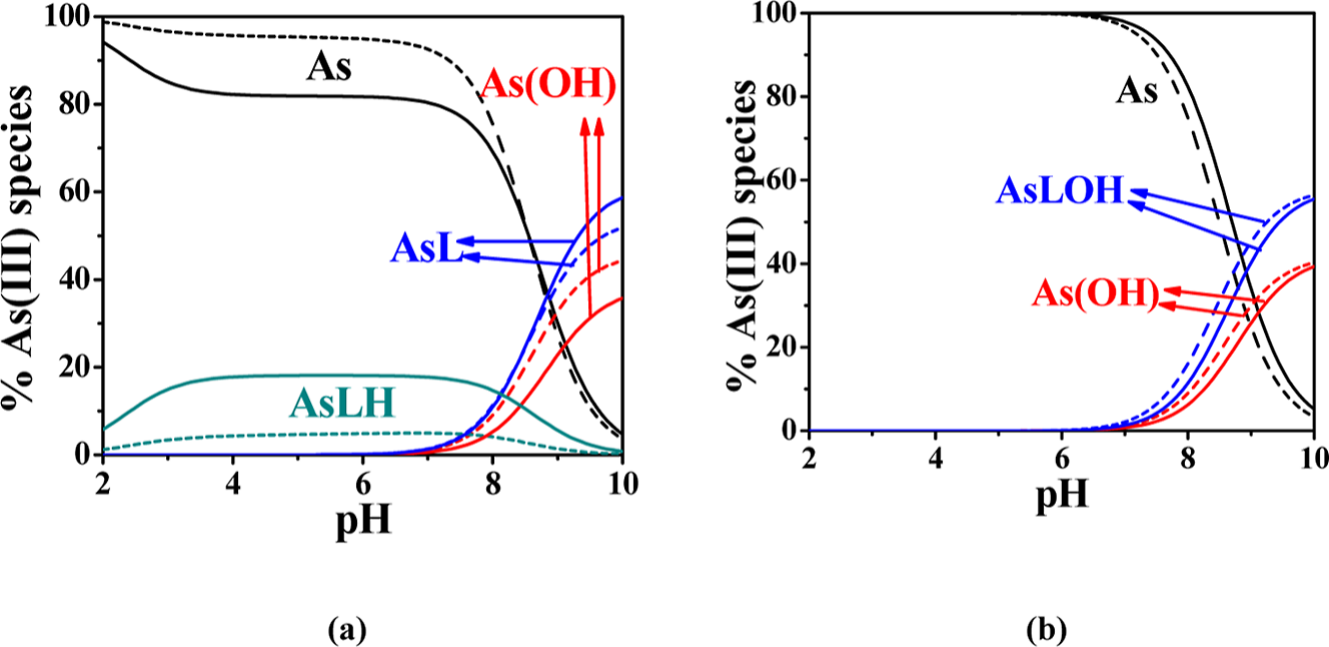
(a) Distribution diagram of As­(III)–MNZ­(L) and (b) As­(III)–NAL­(L) species, at *C*_MNZ_ = 2 mmol L^–1^, *C*_NAL_ = 2 mmol L^–1^, *C*_As_ = 1 mmol L^–1^, *t* = 25°C (solid lines), and *t* = 37°C (dotted lines), *I* = 0.15 mol L^–1^.

To the best of our knowledge, the literature lacks stability data on metal complexes formed by MNZ and NAL with As­(III). The results obtained in this study can be compared with those obtained with the same ligands with different cations. For example, MNZ interacts with Zn^2+^ forming three species, namely, MLH, ML, and MLOH.^55^ The stability of MLH species (log*K* = 1.87) is not too different from that of As­(III) (log*K* = 2.09) under the same conditions (*t* = 25°C, *I* = 0.16 mol L^–1^). Other comparisons with Cu^2+^ e Ca^2+^ are listed in Table S3.^54,55^ The stability of the As­(III)–NAL species can also be compared to that of the complexes containing Fe­(III) and Cr­(III). For these systems, the speciation models reported in the literature are different from As­(III) one and the log*K* values result fairly high, in particular for Fe­(III).^56^ In Table S3, the formation constants of NAL with Mn^2+^ and Zn^2+^ are also listed.

Spectrophotometric titrations were conducted to validate and compare the preliminary potentiometric findings regarding the speciation models and formation constant values. The formation constant values obtained via spectrophotometric titrations for both systems agree with those derived from the potentiometry. In Table 4, the results obtained by both methods were listed together with suggested values at *t* = 25°C and *I* = 0.15 mol L^–1^.

**Table 4:** Comparison between Experimental Formation Constants Obtained by Potentiometry and UV Spectrophotometry, at *t* = 25°C and *I* = 0.15 mol L^–1^

L	species	logβ_UV_ ^a^	logβ_potentiometry_ ^a^	logβ_suggested_ ^a^
MNZ	AsLH	13.76	13.76(10)^b^	13.76(10)^b^
	AsL	5.80(6)^b^	5.63(2)	5.72(6)
NAL	AsLOH	–5.90(6)	–6.13(5)	–6.02(6)

a β refers to the reaction: As + L + *i*H = AsLH_*i*_ (charges omitted for simplicity).

b ≥95% of confidence interval.

Selected spectra at various pH values are presented in the Supporting Information (Figures S1 and S2). Additionally, Figure 3a illustrates the molar extinction coefficients (ε) of each As­(III)–MNZ species relative to wavelength (λ). Both AsL and AsLH species exhibit a maximum peak at λ = 325 nm with coefficients of 12,450 and 12,500 L mol^–1^ cm^–1^, respectively. In Figure 3b, the AsLOH species containing NAL shows two distinct peaks at λ = 250 and 328 nm, with coefficients of 41,500 and 22,500 L mol^–1^ cm^–1^, respectively. The trend of ε vs λ highlights that the formation of AMCs causes both for MNZ and NAL a significant increase in the UV absorption properties of the complexes compared to the precursors of the antibiotics.

**Figure 3:**
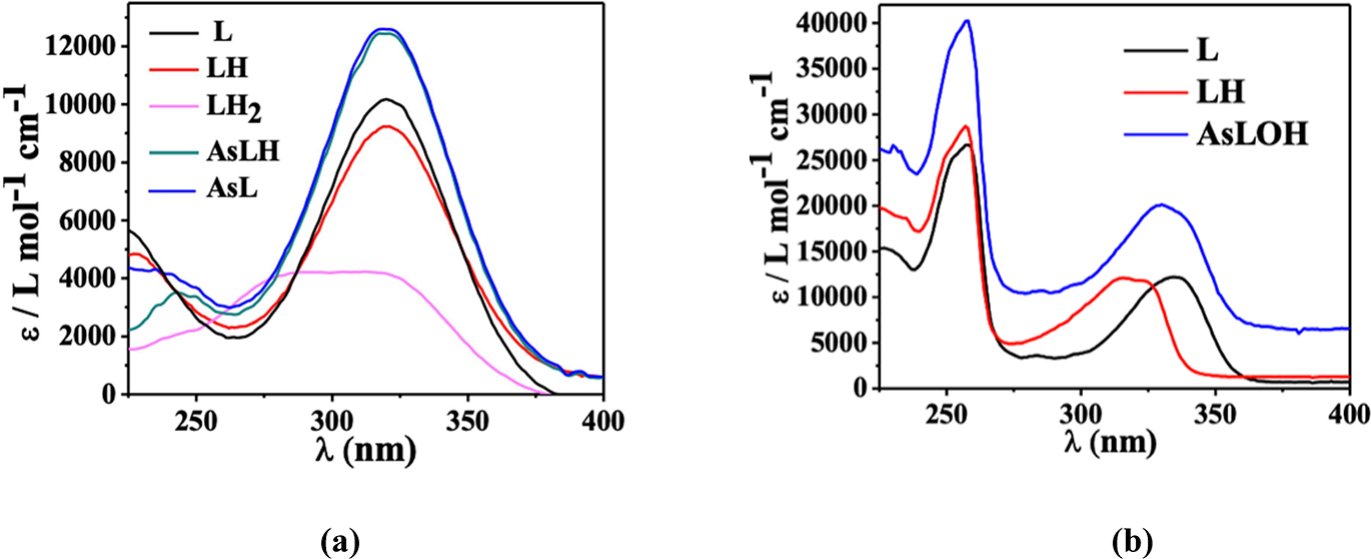
ε vs λ at *t* = 25°C, *I* = 0.15 mol L^–1^ of: (a) As­(III)–MNZ­(L), (b) As­(III)–NAL­(L) species.

### 3.2 Dependence of Formation Constant on Temperature and Ionic Strength

Starting from the knowledge of the formation constant values at different temperatures, the enthalpy change values were calculated by using the following van’t Hoff-type equation^57^

$$\log^{T}\beta ={\log\beta }_{\theta }+\Delta H_{\theta }\left(\frac{1}{\theta }-\frac{1}{T}\right)$$ 1

where log^*T*^β is the equilibrium constant at a specific temperature (Kelvin); θ is the reference temperature (Kelvin); logβ_θ_ is the equilibrium constant at *t* = 298.15 K. The values of formation enthalpy changes of the species of both systems are collected in Table 5, together with entropy and free energy changes. Based on the reported results, the As­(MNZ)H and As­(MNZ) species forms spontaneously (Δ*G* < 0) and exhibit exothermic enthalpy change values.

**Table 5:** Δ*G*, Δ*H*, *T*Δ*S* of As­(III)–MNZ­(L) and As­(III)–NAL­(L) Species at *t* = 25°C and *I* = 0.15 mol L^–1^ in NaCl, Together with Formation Constants at Infinite Dilution and Parameter *C* for the Dependence on the Ionic Strength (eq 2) at *t* = 25°C

L	species^a^	Δ*G* ^b^	Δ*H* ^b^	*T*Δ*S* ^b^	log^*T*^β	*C*
MNZ	AsLH	–78.5(6)^*c*^	–109(5)^c^	–30(5)^c^	13.9(1)^c^	0.98(6)^c^
	AsL	–32.1(1)	–71(1)	–39(1)	6.3(2)	0.8(1)
NAL	AsLOH	35.0(3)	67(10)	32(10)	–6.2(2)	2.6(2)

a Reaction: As + L + *i*H = AsLH_*i*_ (charges omitted for simplicity).

b In kJ mol^–1^.

c ≥95% of confidence interval.

With the aim of evaluating the dependence of the stability constants on ionic strength for all the species of the systems under study, the experimental values obtained in the range of ionic strength between 0.15 and 1 mol L^–1^ were analyzed by means of the following Debye–Hückel-type equation^55^

$$\log\beta ={\log\beta }^{0}-0.51z^{*}\frac{\sqrt{I}}{1+1.5\sqrt{I}}+CI$$ 2

where β^0^ is the constant at infinite dilution, z* is the parameter that refers to the charges of the species involved in the formation equilibrium, i.e., ∑*z*_reagents_^2^ – ∑*z*_products_^2^, and *C* is an empirical parameter that depends on the stoichiometric coefficients and on charges. The resulting parameters are reported in Table 5. The knowledge of the thermodynamic constants and *C* parameters for each species is useful to calculate the stability constants at any ionic strength over the entire investigated range.

The evaluation of the influence of pH, temperature, and ionic strength of the AMCs formation is of considerable importance for the implementation of remediation technologies, such as AOPs, nanomaterials for adsorption or degradation, and bioremediation by microorganisms, to enable the efficient removal of AMCs from contaminated waters.

### 3.3 Sequestering Ability

Both As­(III) and the ligands MNZ and NAL, as highlighted in the introductory paragraph, are persistent pollutants in natural waters, with concentrations reaching up to 3 μg L^–1^ for arsenic (14th most abundant element in seawater), 0.75 μg L^–1^ for NAL, and 0.82 μg L^–1^ for MNZ.^58–60^ The formation of AMCs is expected to further aggravate environmental risks, particularly in aquatic ecosystems, where their accumulation can lead to bioaccumulation and toxic effects on aquatic life. The speciation study presented here is crucial for developing effective systems aimed at removing not only these pollutants but also their associated complex species from natural water systems, thereby mitigating their environmental impact. However, relying solely on formation constant values to predict the behavior of ligands in solution may be insufficient. This is because metal–ligand interactions are influenced by multiple factors, including competing equilibria and the presence of other ions and species in the solution that can alter the stability of the complexes. Thus, a more comprehensive understanding of these interactions, especially under relevant natural water conditions, is particularly important for accurately understanding the capability of those antibiotics to form AMCs. Sequestering ability refers to a ligand’s capacity to form stable complexes with metal cations in solution, thereby reducing the concentration of free metal cations. This process is important for removing toxic metals such as As­(III) from contaminated environments. The sequestering ability of both ligands against As­(III) was evaluated by using the empirical parameter pL_0.5_. This represents the cologarithm of the ligand concentration required to sequester 50% of the trace metal cations present in solution. The pL_0.5_ value is derived from the following Boltzmann-type equation^45^

$$\chi =\frac{1}{1+10^{\left(pL-pL_{0.5}\right)}}$$ 3

where χ is the sum of the mole fractions of the metal–ligand species and pL is the cologarithm of the total ligand concentration. The higher the value of pL_0.5_, the greater the ligand ability to sequester a metal cation in solution as it indicates that a lower concentration of ligand is needed to remove a significant proportion of metal ions. To simulate natural water conditions, the formation constants of the As-L species, as well as the protonation and hydrolytic constants were calculated by eq 2 and were taken into account at *t* = 25°C, pH = 5.0, *I* = 0.001 mol L^–1^ as an example of a fresh water condition and at *t* = 25°C, pH = 8.1, *I* = 0.7 mol L^–1^ as seawater condition.

Based on the reported formation constants (Table 3), MNZ is expected to form more stable complexes than NAL under a variety of temperature and ionic strength conditions. Consequently, it would be expected that MNZ would exhibit a greater sequestering ability toward As­(III) compared to NAL. However, Figure 4a reveals a different trend: under seawater conditions (pH = 8.1, I = 0.7 mol L^–1^, and *t* = 25°C), NAL exhibited a slightly higher sequestering ability (pL_0.5_ = 2.1) than MNZ (pL_0.5_ = 1.7). This deviation from the expected trend highlights the complexity of ligand behavior in real-world environmental conditions, where factors such as the presence of competing ions may influence their effectiveness. Additionally, when comparing the sequestering ability of MNZ toward As­(III) in seawater (pL_0.5_ = 1.7) and an example of river water conditions (pL_0.5_ = 0.4), it is evident that MNZ exhibits a much higher ability to sequester As­(III) under seawater conditions (Figure 4b). This suggests that the higher ionic strength and composition of seawater enhance the MNZ ability to form complexes with As­(III), further emphasizing the importance of considering the specific characteristics of the water matrix when assessing a ligand performance.

**Figure 4:**
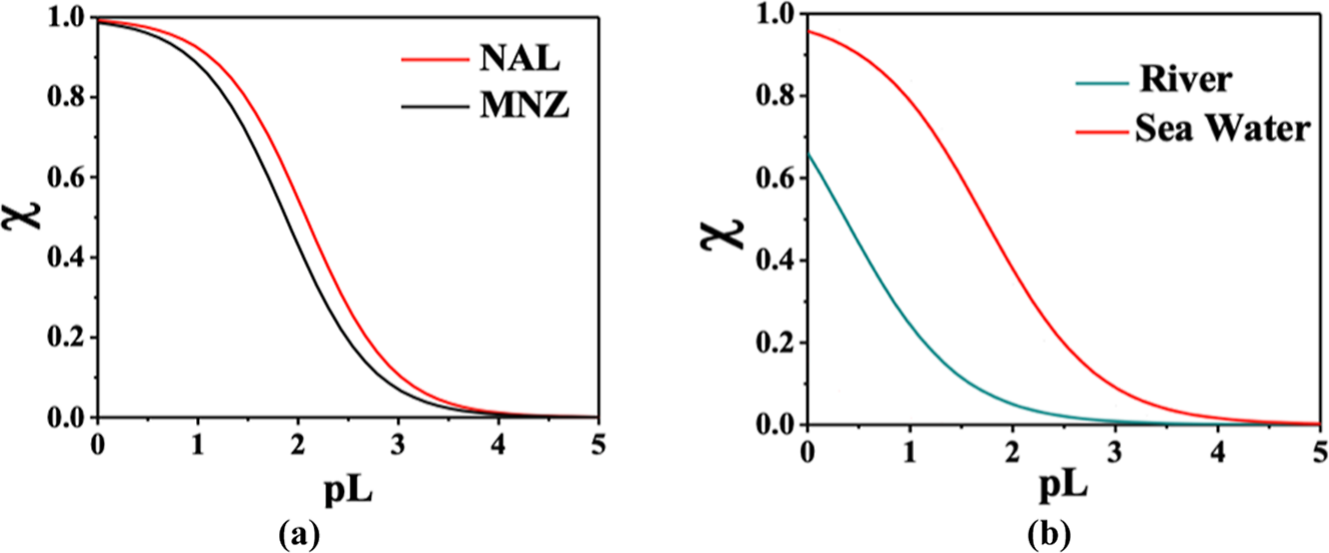
(a). Sequestering abilities of MNZ and NAL toward As­(III) under seawater conditions (pH = 8.1, *I* = 0.7 mol L^–1^, *t* = 25°C). (b) Sequestering ability of MNZ toward As­(III) under both seawater (pH = 8.1, *I* = 0.7 mol L^–1^, *t* = **25 °C**) and river water (pH = 5.0, *I* = 0.001 mol L^–1^, *t* = 25°C) conditions.

The results underscore the necessity of evaluating a broader range of environmental variables such as solution pH, ionic strength, and the presence of competing species when assessing the sequestering ability of ligands. These variables can significantly influence the stability of metal–ligand complexes and, consequently, the effectiveness of a ligand in metal remediation. The observed trends challenge the assumption that the stability of metal–ligand complexes, as predicted by formation constants, directly correlates with the sequestering ability in natural systems. Instead, these findings highlight the complexity of environmental systems where multiple interacting factors must be considered.

### 3.4 Quantum-Mechanical Calculations

With the aim of investigating the binding capabilities of the MNZ and NAL species toward the As­(III) moiety in solution, a series of DFT calculations were conducted. Among the possible chelation sites where some [As­(OH)_*x*_]^*y*^ (*x* = 1,2; *y* = 2+,1+) species can be hosted in the MNZ structure, two specific sites exhibited a measurable propensity toward binding, namely, the nitro-functional group and the hydroxyl group. In the case in which the binding occurs at the nitro group, several molecular configurations of possible [MNZ­(H) + As­(OH)_*x*_]^*y*^ complexes were simulated at the B3LYP/6–311++G­(d,p) theory level using the CPCM implicit water solvation model. The two structures displayed in Figure 5 emerged as the most stable. In fact, whilst the formation of the [MNZ + As­(OH)]^2+^ complex with the [As­(OH)]^2+^ moiety chelated by the nitro-functional group led to unstable molecular structures, the complexes [MNZ + As­(OH)_2_] (i.e., As­(MNZ)) and [MNZH + As­(OH)_2_]^+^ (i.e., As­(MNZ)­H) depicted in Figure 5a,b are characterized by significant binding energies of −32.8 and −29.6 kcal/mol, respectively. This way, although the binding occurs at the same site, slight but measurable differences in the interaction strength are induced by a deeply different electronic structure in the two structures. The optimized ML molecular structure displayed in Figure 5a is neutral, while the MLH structure shown in Figure 5b is positively charged. Notwithstanding the marginally different binding energy values, the local stable arrangement of the atomic species featuring the overall geometry of the two complexes is essentially identical, a circumstance indicating that the two complexes can be easily interconverted into each other depending on the external pH conditions. In fact, both complexes exhibit a very similar As­(III)–N interaction featured by an equilibrium length of ≈2.04–2.05 Å.

**Figure 5:**
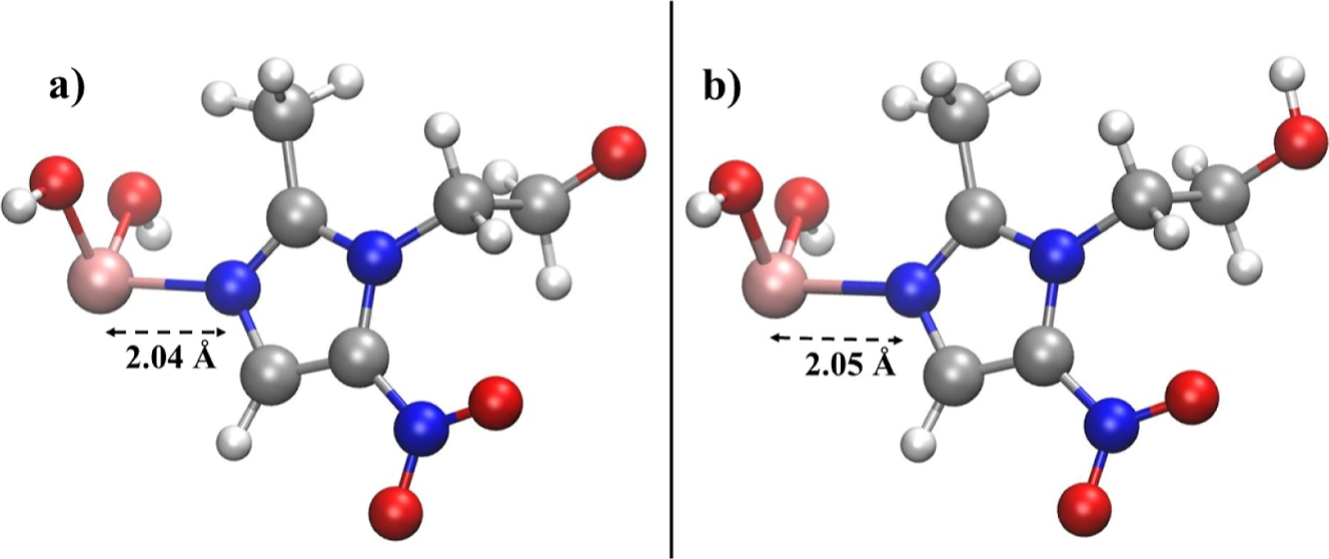
Molecular structure of the complexes As­(MNZ) (a) and As­(MNZ)H (b) formed by the direct one-to-one binding at the nitro-functional group of As­(III) with MNZ optimized at the B3LYP/6–311++G­(d,p) theory level under the CPCM implicit water solvation model. Equilibrium As­(III)–N distances are shown in Å.

Drastically different is the situation for the As­(MNZ) and As­(MNZ)­H structures formed when the binding takes place at the hydroxyl group. In fact, the neutral complex [MNZ + As­(OH)_2_] displayed in Figure 6a turns out to be significantly more stable with respect to its positively charged counterpart [MNZH + As­(OH)_2_]^+^ shown in Figure 6b. In particular, although the former is characterized by a large binding energy of −75.8 kcal/mol, the [As­(OH)_2_]^+^ moiety binds the hydroxyl group of the MNZH molecule with a significantly lower strength of −16.3 kcal/mol. Such energetic evidence is further magnified by monitoring the equilibrium distance between the O atom of the hydroxyl group and the approaching As­(III) one. When the oxygen atom is deprotonated, a very strong interaction in the As­(MNZ) complex is witnessed by a short As­(III)–O interatomic equilibrium distance of only 1.80 Å (Figure 6a), which increases up to 2.13 Å in the case of the As­(MNZ)H structure (Figure 6b).

**Figure 6:**
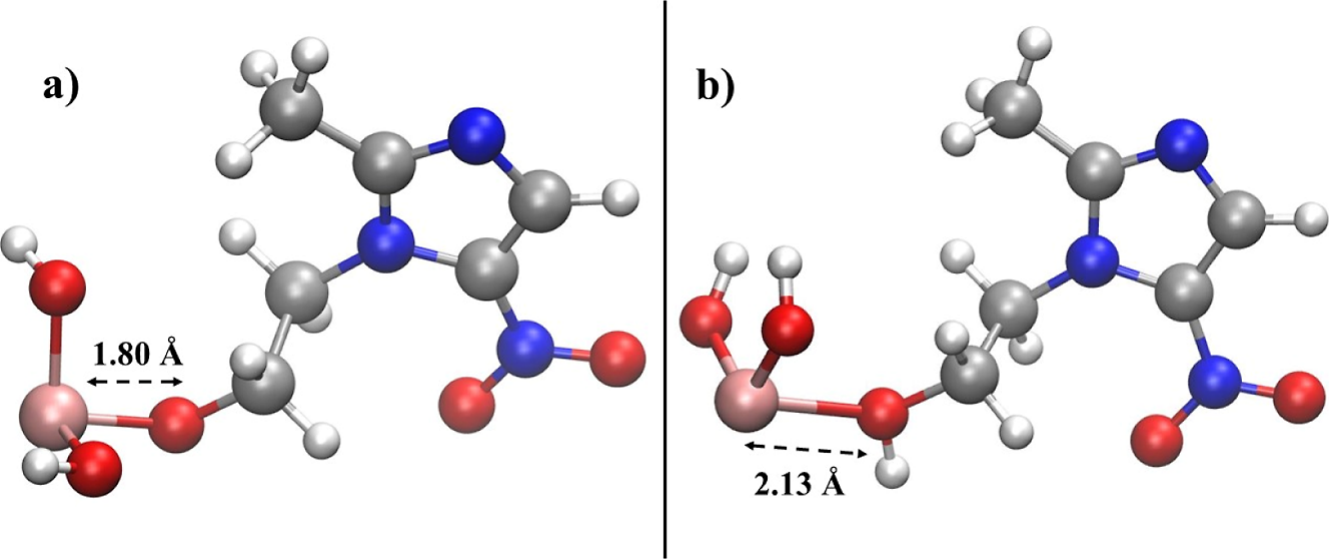
Molecular structure of the complexes As­(MNZ) (a) and As­(MNZ)H (b) formed by the direct one-to-one binding at the hydroxyl group of As­(III) with MNZ optimized at the B3LYP/6–311++G­(d,p) theory level under the CPCM implicit water solvation model. Equilibrium As­(III)–O distances are shown in Å.

Incidentally, by comparing the absolute energy values in structures belonging to the same thermodynamical ensemble (*i.e*., having the same number of atoms and the same global charge), it turns out that while among the As­(MNZ) moieties the binding preferentially occurs at the hydroxyl site (Figure 6a), among the As­(MNZ)H structures, the bonding takes place at the nitro-functional group (Figure 5b). All these findings are consistent with the speciation curve reported in Figure 2a, where the positively charged As­(MNZ)­H species dominates under acidic conditions, but at slightly basic pH values (≈8), it becomes thermodynamically unfavored with respect to its neutral AsMNZ counterpart—and specifically the complex shown in Figure 6a—which becomes strongly favored by virtue of a very large binding energy with [As­(OH)_2_]^+^.

It is worth noting that both the estimates of the absolute value of the binding energies and the optimized As­(III)–N/O distances might be improved by adopting higher-level DFT computations. On the other hand, the relative stability of the complexes should not be that affected by the specific choice of the DFT framework, provided that sufficiently large basis sets and adequate exchange and correlation functionals are employed. Furthermore, as also proven by our group in several studies,^61–63^ it is well-known that an explicit treatment of the solvent might lead to very different binding modalities.

Similarly to the MNZ species, the deprotonated NAL structure offers at least two quite promising sites for the interaction with the partially hydrated As­(III) cation. However, the carboxylic group was not capable of forming stable molecular arrangements with the [As­(OH)]^2+^ species, likely due to steric hindrance. For the same reason, the potentially stabilizing interaction between [As­(OH)_2_]^+^ and the nitrogen atom of the pyridine ring did not lead to any energetically favorable molecular configuration.

Beyond the just mentioned possibilities, a very stable complex can certainly be formed between the [As­(OH)]^2+^ species and the deprotonated NAL molecule, as shown in Figure 7. In particular, the electron density around one of the oxygen atoms of the carboxylic group in position 3 and the oxygen atom of the carbonyl group in position 4 in conjunction with the natural volume of the respective nuclei create the goldilocks conditions for chelation. This way, a very strong interaction is triggered with the [As­(OH)]^2+^ moiety and the very stable [NAL + As­(OH)]^+^ complex is formed accompanied by a huge energetical gain of −160.1 kcal/mol, a value testifying once again the thermodynamic favorability of such a complex. The literature confirms this kind of coordination for the class of quinolones to which NAL belongs. More in detail, usually the quinolones act as a bidentate ligand, via an oxygen atom of the carboxylic group and another oxygen atom of the carbonyl group on the ring.^64,65^ Incidentally, the two interactions that As­(III) establishes with the O atom of the carboxylic group and the O atom of the CO group are characterized by an equilibrium reciprocal length of 1.85 and 1.89 Å, respectively. The distance values show the strength of the interactions and the stability of the whole complex.

**Figure 7:**
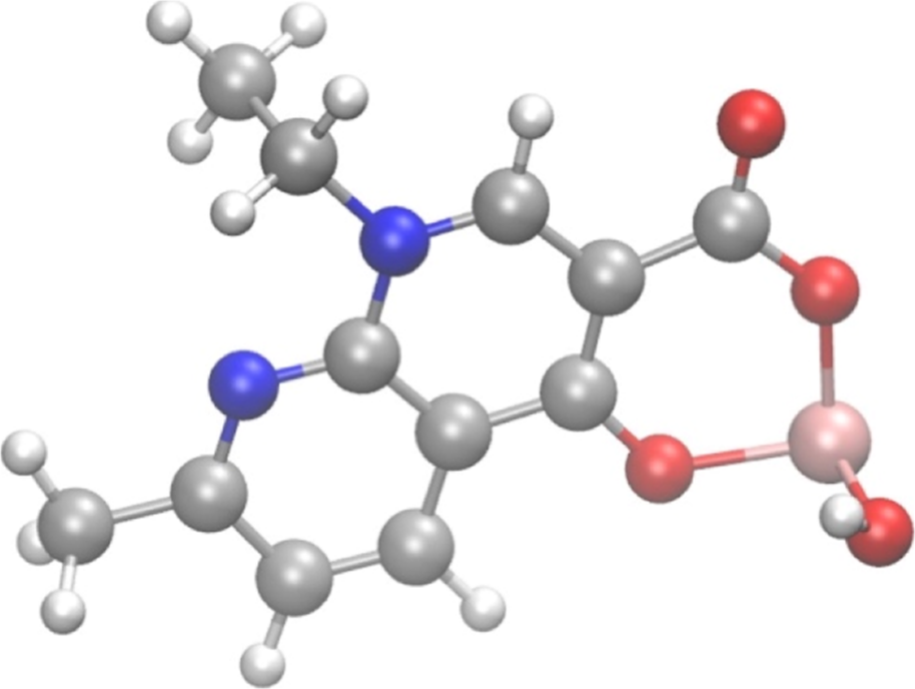
Molecular structure of the complex formed by the direct one-to-one binding of As­(III) with NAL­(L) optimized at the B3LYP/6–311++G­(d,p) theory level under the CPCM implicit water solvation model.

## 4 Conclusions

In this study, the interaction between As­(III) and the antibiotics MNZ and NAL in aqueous solutions has been comprehensively explored, yielding interesting insights into the formation and stability of their respective complexes. Potentiometric and UV spectrophotometric titrations revealed that MNZ forms both AsL (pH ≥ 7.5) and AsLH (2 ≤ pH ≤ 10) complexes, while NAL forms only the AsLOH at pH ≥ 7.5. The thermodynamic data indicate that the formation of the As­(III)–MNZ complexes is spontaneous and energetically favorable. In addition, it turns out that the sequestering ability of MNZ and NAL toward As­(III) varies significantly across different water matrices, with NAL showing a slightly higher sequestration ability in seawater compared with MNZ. This highlights the complexity of ligand behavior in natural waters, where factors such as pH and ionic strength influence the efficiency of metal sequestration strategies.

DFT calculations further elucidated the structural interactions, showing that MNZ binds to As­(III) through both As–N and As–O interactions in its neutral (AsMNZ) or protonated (As­(MNZ)­H) forms, while NAL forms a stable chelated complex via bidentate coordination. Our computational models also reveal that, for the AsMNZ complex, the oxygen atom of the hydroxyl group is the most favorable binding site for As­(III), while both the nitro- and hydroxyl groups are similarly efficient in the As­(MNZ)H complex.

These findings are significant because they provide a detailed understanding of the speciation and stability of As­(III)–antibiotic complexes, which are crucial for developing targeted strategies for mitigating arsenic contamination. Specifically, a deeper understanding of the speciation of As­(III)-ligand complexes as a function of factors like temperature, ionic strength, and pH is crucial for identifying the optimal conditions for the decomplexation process (e.g., increasing the temperature to *t* = 37 °C or higher for As­(III)–MNZ and lowering the pH for As­(III)–NAL), thereby improving remediation techniques.

Future research will focus on investigating the interaction between As­(III) and antibiotics in the presence of key components typically found in real aquatic environments (e.g., Na^+^, Cl^–^, Mg^2+^, Ca^2+^, K^+^, Fe^2+^, and Fe^3+^), where the variable presence of competing ions can influence the complex behavior. Investigating these systems under more realistic conditions yields critical insights into pollutant dynamics, thereby guiding the design of more robust, adaptable, and efficient water treatment approaches.

## Supplementary Material



## Notes

Protonation constant values of MNZ and NAL species at different temperatures and ionic strength values; hydrolysis constant values of As­(III) species at different temperatures and ionic strength values; formation constants at *t* = 25 °C and *I* = 0.15 mol L^–1^; UV spectra at selected pH values of As­(III)–MNZ solution; and UV spectra of As­(III)–NAL­(L) solution at selected pH values (PDF)

## Abbreviations

MNZ: metronidazole
NAL: nalidixic acid
L: ligand
AMCs: antibiotic–metal complexes
CEC: contaminants of emerging concern
