# Supplementary material for: Speciation and Thermodynamic Study of Arsenic(III)–Pharmaceutical Complexes in Aqueous Solutions
Source: ACS Environ Au. 2025 May 12;5(4):404–14. doi: 10.1021/acsenvironau.5c00024 (PMC12272278; doi:10.1021/acsenvironau.5c00024)
Supplement: Supplementary file 1 [file vg5c00024_si_001.pdf]

Speciation and Thermodynamic Study of Arsenic(III) – Pharmaceutical Complexes in Aqueous Solutions

Federica Carnamucio[a]*, Claudia Foti[b], Franz Saija[c], Giuseppe Cassone[c], Ottavia Giuffrè[b,c]*

[a] Department of Pharmaceutics and Center for Pharmaceutical Engineering and Sciences - School of Pharmacy, Virginia Commonwealth University, 410 N 12th St. Richmond, 23284 Virginia (United States).

[b] Dipartimento di Scienze Chimiche, Biologiche, Farmaceutiche ed Ambientali, Università di Messina, Viale F. Stagno d'Alcontres 31, 98166 Messina (Italy).

[c] Institute for Chemical-Physical Processes, National Research Council of Italy (CNR-IPCF), Viale Ferdinando Stagno d'Alcontres 37, 98158 Messina (Italy).

*Emails: FC carnamuciof@vcu.edu, OG ogiuffre@unime.it

Table S1. Protonation constant values of MNZ and NAL species at different temperatures and ionic strength values.

Ligand	Reaction	t /°C	I / mol L^{-1}	logβ
MNZ	$L^- + H^+ = LH^0$	15	0.15	12.00 [1]
		25	0.15	11.674
		25	0.5	12.51
		25	1	12.22
		37	0.15	11.88
	$L^- + 2H^+ = LH_2^+$	15	0.15	14.559 [1]
		25	0.15	14.130
		25	0.5	15.18
		25	1	15.05
		37	0.15	14.27
NAL	$L^- + H^+ = LH^0$	15	0.15	6.025 [2]
		25	0.15	5.946
		25	0.5	5.809
		25	1	5.705
		37	0.15	5.82

[1] F. Carnamucio, C. Foti, M. Cordaro, O. Giuffrè. Study on Metronidazole Acid-Base Behavior and Speciation with Ca^{2+} for Potential Applications in Natural Waters. Molecules, 2022, 27(17), 5394.

[2] F. Carnamucio, D. Aiello, C. Foti, A. Napoli, O. Giuffrè, Aqueous chemistry of nalidixic acid and its complexes with biological relevant cations: A combination of potentiometric, UV spectrophotometric, MS and MS/MS study, J of Inorg Biochem, 2023, 249, 112366.

Table S2. Hydrolysis constant values of As(III) species at different temperatures and ionic strength values.

**Reaction**	**t /°C**	**I / mol L^{-1}**	**logβ[1]**
$As(OH)_3 + H_2O = As(OH)_4^- + H^+$	15	0.15	-9.26
	25	0.15	-9.12
	25	0.5	-9.03
	25	1	-9.02
	37	0.15	-8.92

[1] G. Cassone, D. Chillè, C. Foti, O. Giuffrè, R. C. Ponterio, J. Sponer, F. Saija, Stability of hydrolytic arsenic species in aqueous solutions: As^{3+} vs As^{5+}, Phys. Chem. Chem. Phys., 2018, 20, 23272.

Table S3. Formation constants at t = 25°C and I = 0.15 mol L^{-1}.

Ligand	Species	**log*K*** [1]				
		M = Zn(II) [2]	M = Cu(II) [2]	M = Ca(II) [3]	M = As(III) [4]	
MNZ	MLH	1.87	–	2.49	2.09	
	ML	6.57	8.87	–	5.63	
	ML_2	–	8.52	–	–	
	MLOH	-8.71	–	–	–	
		**log*K***				
Ligand	Species	M = Zn(II) [5]	M = Mn(II) [5]	M = As(III) [4]	M = Fe(III)[5,6]	M = Cr(III)[5,6]
NAL	ML	3.47	4.25	–	–	9.23
	ML_2	–	–	–	7.46	5.46
	ML_3	–	–	–	4.99	3.65
	MLOH	3.94	–	2.97	–	–

[1] *K* refers to the reaction: As + LH_i = $AsLH_i$ (charges omitted for simplicity); [2] Carnamucio, F.; Foti, C.; Micale, N.; Van Pelt, N.; Matheeussen, A.; Caljon, G.; Giuffrè, O. Metronidazole Interaction with Cu $^{2+}$ and Zn $^{2+}$: Speciation Study in Aqueous Solution and Biological Activity Evaluation. *ACS Omega* **2024**, *9* (26), 29000–29008. [3] Carnamucio, F.; Foti, C.; Cordaro, M.; Giuffrè, O. Study on Metronidazole Acid-Base Behavior and Speciation with Ca^{2+} for Potential Applications in Natural Waters. *Molecules* **2022**, *27* (17), 5394; [4] this paper; [5] citare lavoro Molecules 2023; [5] Farghaly, O. A.; Al–Saidi, H. M.; Naggar, A. H.; El–Mabrouk, I. M. Metal Complexes and Determination of Nalidixic Acid by Potentiometric and Conductometric Methods. *Int. J. Electrochem. Sci.,* **2017***, 12,* 9865 – 9881*;* [6] I = 0.01 mol L^{-1}.

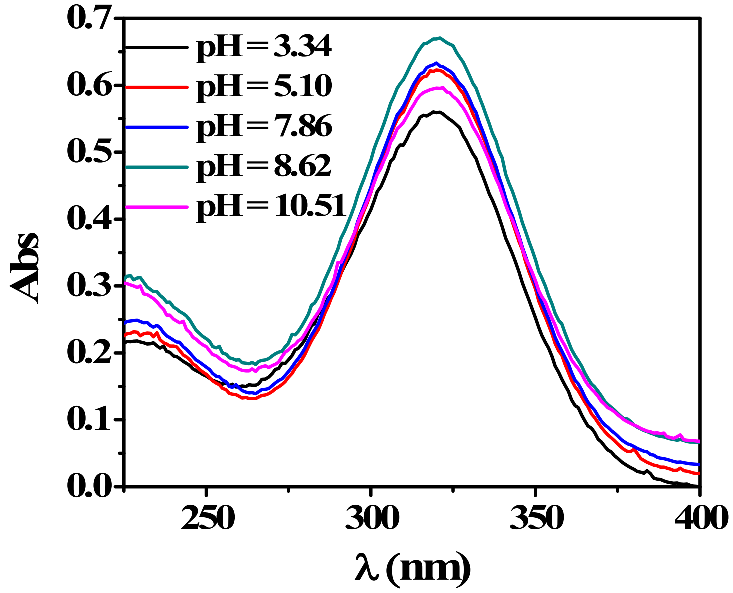

Figure S1. UV spectra at selected pHs of As(III)-MNZ solution, $C_{As} = C_{MNZ} = 0.05$ mmol L^{-1}, at t = 25°C, I =0.15 mol L^{-1}.

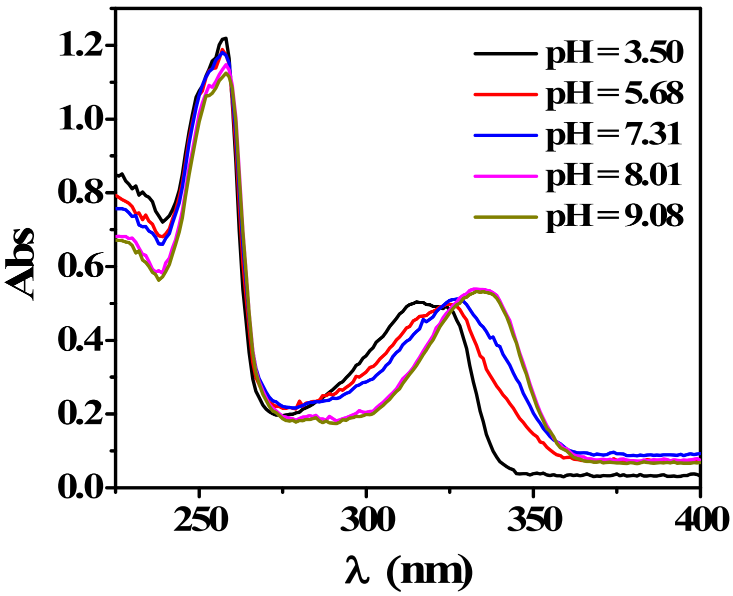

Figure S2. UV spectra of As(III)-NAL(L) solution, t = 25°C, I = 0.15 mol L^{-1}, C_M = C_{NAL} = 0.04 mmol L^{-1}.
